# Bacteriophage-Mediated Perturbation of Defined Bacterial Communities in an *In Vitro* Model of the Human Gut

**DOI:** 10.1128/spectrum.01135-22

**Published:** 2022-05-31

**Authors:** Hedieh Attai, Jacob Wilde, Roland Liu, Jessica Chopyk, Andrew G. Garcia, Emma Allen-Vercoe, David Pride

**Affiliations:** a Department of Pathology, University of California, San Diego, La Jolla, California, USA; b Department of Molecular and Cellular Biology, University of Guelph, Guelph, Ontario, Canada; c Department of Medicine, University of California, San Diego, La Jolla, California, USA; University of Pittsburgh School of Medicine

**Keywords:** phage perturbations, bacteriophages, phages, *in vitro* communities, robogut, virus transfers

## Abstract

The study of bacteriophage communities reproducing in the gastrointestinal tract is limited by the quality of model systems supporting experimental manipulation *in vitro*. Traditionally, studies aiming to experimentally address phage-bacteria dynamics have utilized gnotobiotic mice inoculated with defined bacterial communities. While mouse models simulate complex interactions between microbes and their host, they also forestall the study of phage-bacteria dynamics in isolation of host factors. Here, we established a method for manipulating phage-bacteria dynamics using an *in vitro* chemostat bioreactor model of the distal human gut. We create defined communities representing a subset of bacteria in the feces of two human individuals, cultivated these communities in chemostat bioreactors, developed methods to purify the autochthonous viromes associated with each cultured community, and trialed a system for transmitting live or heat-killed viruses between chemostat bioreactors to decipher outcomes of virus-mediated perturbation. We found that allochthonous viromes were detectable via metagenomic sequencing against the autochthonous virome background and that shifts in bacterial community diversity and composition were detectable in relation to time posttreatment. These microbiome composition changes spanned multiple phyla, including *Bacteroidetes*, *Firmicutes*, and *Actinobacteria*. We also found that compositional changes occurred when using live viruses regardless of whether intrasubject or intersubject viruses were used as the perturbation agents. Our results supported the use of chemostat bioreactors as a platform for studying complex bacteria-phage dynamics *in vitro*.

**IMPORTANCE** Bacteriophages are relatively ubiquitous in the environment and are highly abundant in the human microbiome. Phages can be commonly transmitted between close contacts, but the impact that such transmissions may have on their bacteria counterparts in our microbiomes is unknown. We developed a chemostat cultivation system to simulate individual-specific features of human distal gut microbiota that can be used to transmit phages between ecosystems and measure their impacts on the microbiota. We used this system to transfer phage communities between chemostats that represented different human subjects. We found that there were significant effects on overall microbiota diversity and changes in the relative abundances of *Bacteroidetes*, *Firmicutes*, and *Actinobacteria*, when intersubject perturbations were performed, compared to intrasubject perturbations. These changes were observed when perturbations were performed using live phages, but not when heat-killed phages were used, and they support the use of chemostat systems for studying complex human bacteria-phage dynamics.

## INTRODUCTION

The recent surge of research into the human gut microbiome has brought to light a critical finding: diverse and copious populations of bacteriophages are intimately linked with the structure and function of gut bacterial communities (1–3). Bacteriophages, or phages, are bacterial viruses. They are believed to influence human gut microbiomes by killing their bacterial hosts (1, 4), encoding fitness factors that benefit bacterial hosts (5, 6), and/or facilitating horizontal gene transfer (7). Because the bacterial portion of the gut microbiome is linked to a range of functions integral to host fitness (8–10), phage-bacteria dynamics are expected to have substantial impacts on human health.

Bacteriophages are expected to play a central role in shaping gut microbiome composition and function, just as they do in soil and aquatic ecosystems (11, 12), yet the specific boundaries and parameters of phage influence in the gut remain poorly understood. This lack of understanding *in vivo* is predicated by a lack of understanding *in vitro*. Few model systems exist allowing for experimental exploration of gut phage-bacteria dynamics. Most prior studies of gut bacteriophage populations have relied on metagenomic ‘snapshots’ of fecal samples taken from live mammalian hosts (2, 3, 13, 14). They do not involve experimental control or manipulation. Over the last decade, these snapshots have revealed several interesting phenomena related to gastrointestinal phage communities, including enrichment with temperate phages (3, 14), presence of gut-specialized phage taxa (15), and phage-encoded fitness factors that interact with mammal-encoded factors (5, 6). If researchers are to begin understanding how these phenomena occur and why they have evolved in gastrointestinal ecosystems, platforms that enable experimental manipulation must be developed and trialed.

In the present study, we established a methodology for perturbing complex gut-associated bacterial communities with bacteriophages *in vitro*. To do so, we created defined bacterial communities representing a subset of fecal microbes from two donor individuals, cultivated these communities in chemostat bioreactors, developed methods to purify the autochthonous viromes associated with each cultured community, and trialed a system for transmitting live or heat-killed viruses between chemostat bioreactors to decipher outcomes of virus-mediated perturbation. We evaluated the transmission success of phage-mediated perturbation and analyzed outcomes on microbial community composition and diversity.

## RESULTS

**FIG 1:**
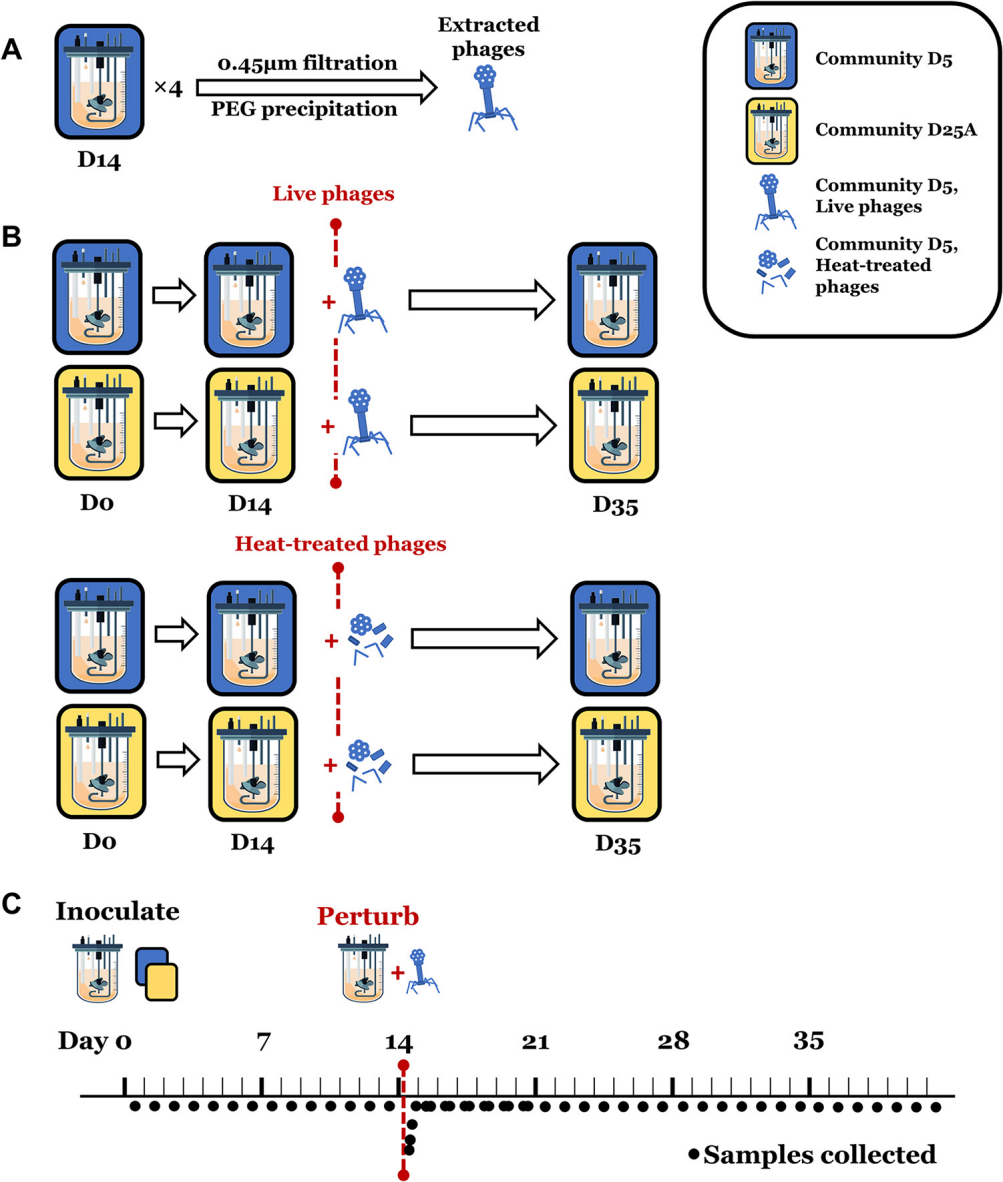
Experimental design and sampling schedule. (A) Four bioreactors were inoculated with community D5. Bioreactor contents were harvested on day 14 and day 21, and purified viruses were pooled. (B) Phage-mediated perturbation experiment: two of four bioreactors were inoculated with community D5 (blue) or community D25A (yellow). After allowing a 14-day equilibration, bioreactors were perturbed with live or heat-treated D5 phage stocks from (A). Following perturbation, bioreactors were allowed to run another 21 days. (C) Timeline of sample collection for each bioreactor in (B). More detailed time points are included in Table S2.

We characterized the outcomes of phage-mediated perturbation on intrahost (autochthonous) or interhost (allochthonous)-defined bacterial communities. Community D5 (77 strains) and community D25A (68 strains) were each used to inoculate one of two chemostat bioreactors. Bioreactor parameters were set to mimic the physiological conditions of the human colon, as in McDonald et al. (16). After allowing the communities 14 days to equilibrate, each was perturbed with a solution of phage particles isolated via 0.45 μm filtration and polyethylene glycol (PEG) precipitation from the effluent of a previous D5 bioreactor run. The second pair of bioreactors were also inoculated with D5 and D25A communities. After 14 days, they received a heat-treated version of the same phage stocks used for the first pair. Experimental design and sample collection are summarized in Fig. 1. Before and at several time points following sample collection, viromes were isolated and sequenced via Illumina short-read sequencing, and bacterial community composition was analyzed through 16s rRNA amplification and sequencing.

### Sequencing output.

After bioreactor sampling, DNA extraction, 16S rRNA sequencing, and quality processing, 123 samples were included in downstream 16S rRNA analyses – 31 representing separate time points in bioreactor conditions D5-live virus (LV), D25A-LV, and D25A-heat-treated virus (HTV), and 30 representing separate time points in condition D5-HTV. In total, 5,664,282 16S rRNA gene sequence reads were included in downstream analyses, with an average of 46,429 (±16,001 SD) reads per sample. To account for unequal sampling depths, data were normalized to equivalence at 16,900 reads per sample.

After bioreactor sampling, virion purification, DNA extraction/amplification, metagenomic sequencing, and quality processing, 56 samples were included in downstream virome analyses –representing 14 time points in bioreactor conditions D5-LV, D5-HTV, D25A-LV, and D25A-HTV. Across all samples, 25,622,997 sequence reads were included in downstream analyses, with an average of 460,779 (±130,505 SD) reads per sample after trimming. Overall, 93.9% (±2.2% SD) of reads were used in contig generation, generating an average of 1,001 (±989 SD) contigs with an average length of 740 (±137.6 SD) base pairs per contig. Complete contig generation metrics are available in Table S1A. Three PEG-purified viromes used for perturbation from replicate bioreactors were sequenced.

### Characterization of viral particles in community D5 bioreactor effluent.

**FIG 2:**
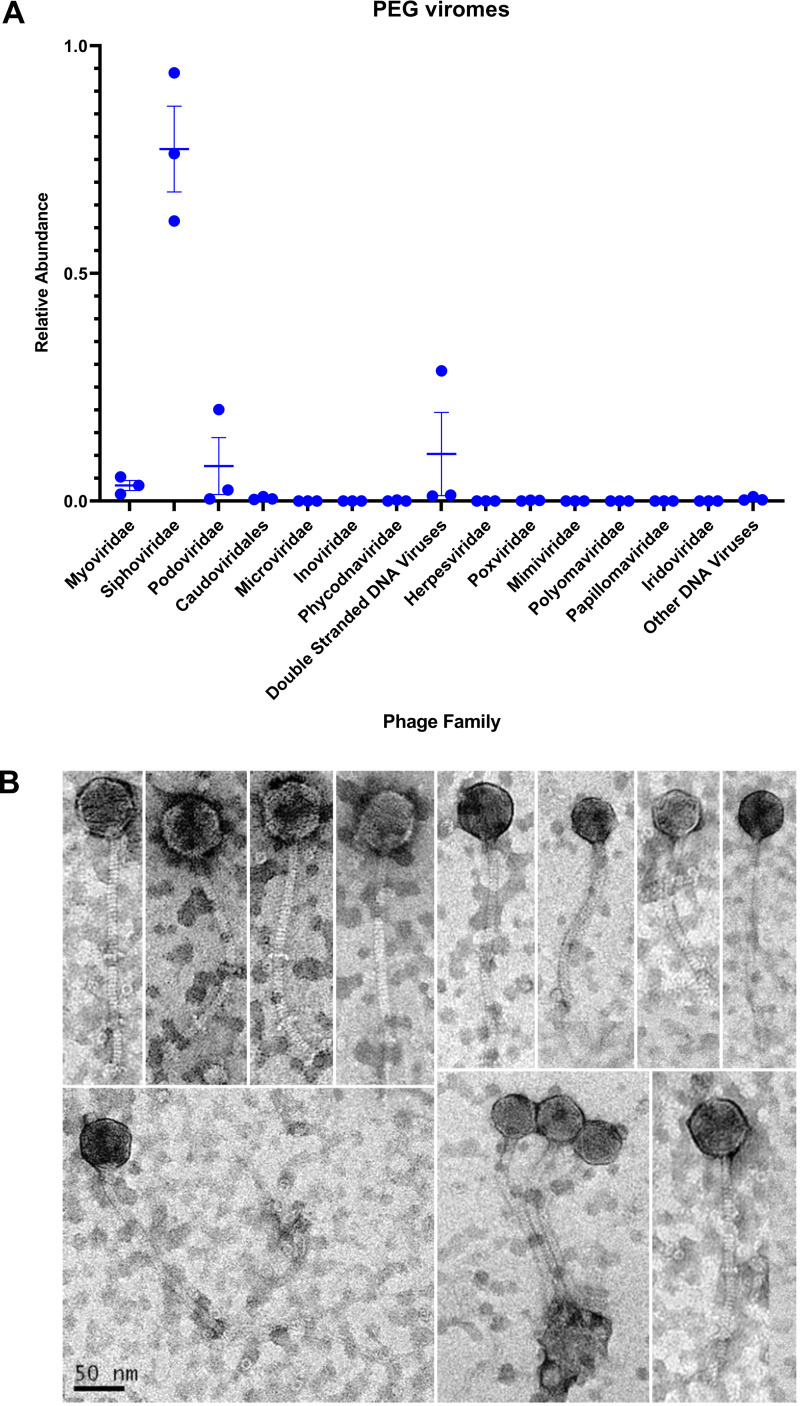
Taxonomic assignment and transmission electron microscopy (TEM) imaging of viruses from D5 bioreactors. (A) Relative abundance of D5 viral contigs by assigned taxonomy. PEG-purified viruses were extracted and sequenced in triplicate. Bars represent mean and standard error. (B) Electron micrographs of representative viruses from D5 bioreactor effluent.

Before their use in perturbation experiments, a fraction of the PEG-purified D5 viruses were characterized by metagenomic sequencing. To complement these data, a fraction of D5 viruses was further purified on cesium chloride density gradients for transmission electron microscopy (6) imaging. Taxonomic assignment of contigs (see Materials and Methods) revealed a prevalence of phages belonging to the order *Caudovirales* (88.4%) and family *Siphoviridae* (77.3%) (Fig. 2A). The remainder of the contigs were homologous to various double-stranded DNA viruses and hereby referred to as ‘unknown double-stranded DNA viruses.’ No representatives of single-stranded DNA or eukaryote-associated viruses were detected. Transmission electron microscopy (TEM) imaging of a CsCl-purified fraction depicted a virome dominated by members of the family *Siphoviridae*, corroborating the taxonomic assignments (Fig. 2B).

### Detection of D5 viruses following their addition to D25A bioreactors.

**FIG 3:**
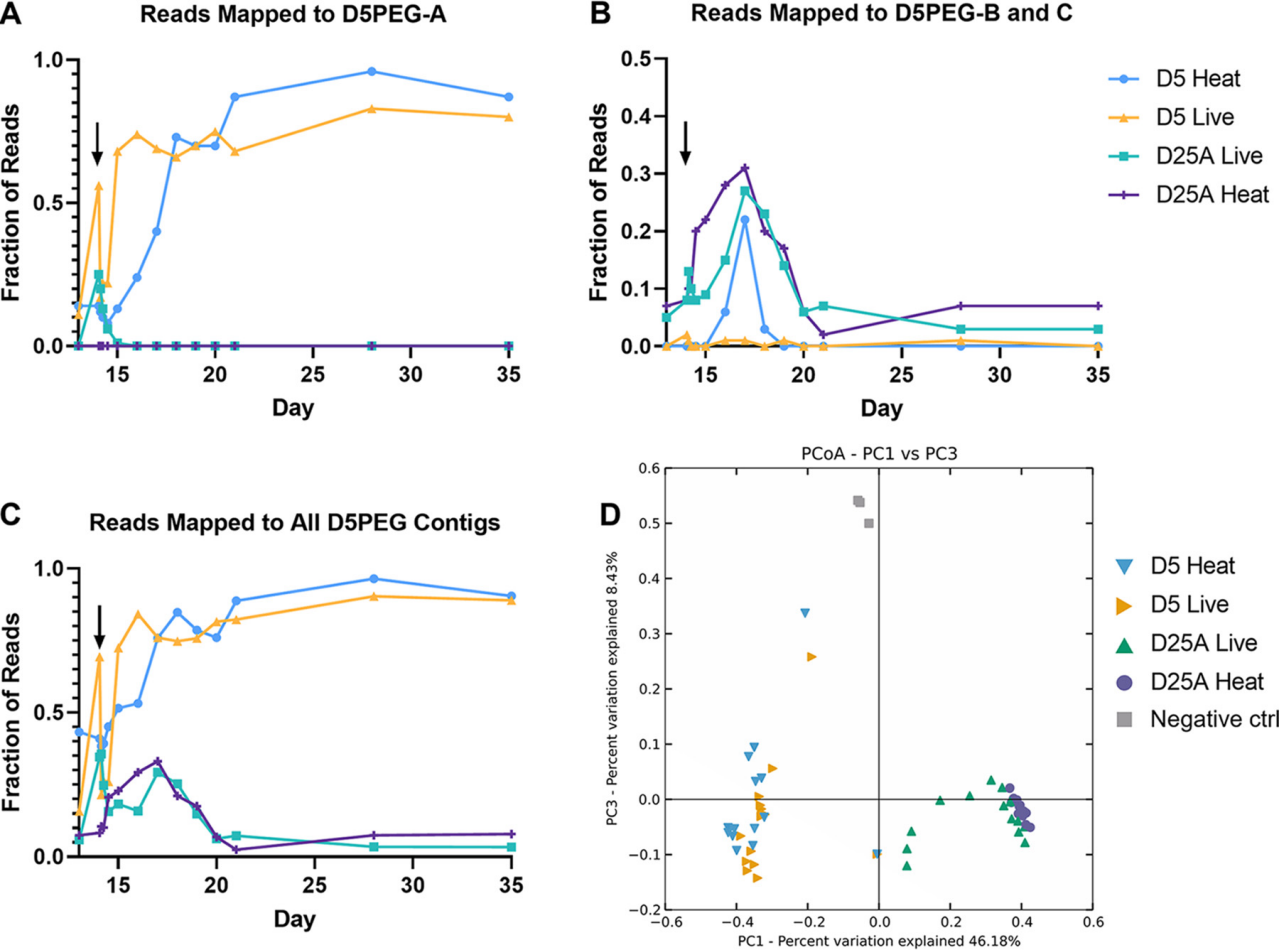
(A to C) Fraction (1 = 100%) of trimmed virome sample sequencing reads that map to (A) contig D5PEG-A, (B) contig D5PEG-B and C, and (C) contigs D5PEG-A, B, and C (Table S1B). The black arrow represents perturbation with D5 PEG-purified viromes on day 14. Before perturbation samples are plotted as day 13 to enable visualization of data. (D) Principal coordinate analysis of virome samples, displaying distances between treatment conditions.

We first sought to determine whether the D5 viruses introduced to the experimental bioreactors could be detected by metagenomic sequencing and whether pretreatment of the D5 viruses with heat and DNase I was effective at rendering the viruses inviable and degrading their genetic material. To determine whether D5 viruses could be detected following perturbation, we mapped reads from perturbed viromes to the three most abundant contigs in the D5 virome used for perturbation (Table S1B). These three contigs were first aligned by nucleotide BLAST against the NCBI nt database. Contig D5PEG-A exhibited high similarity to a *Bacteroides* phage, while contigs D5PEG-B and D5PEG-C exhibited high similarity to the same *Clostridium* phage, suggesting they represent portions of the same bacteriophage genome. Directly following perturbation, we observe a spike in the percentage of reads mapping to the three D5 contigs (Fig. 3A and B) as well as to all the D5PEG virome contigs (Fig. 3C). This spike occurs in the D5 LV and D25A LV bioreactors but not in the bioreactors perturbed with heat-treated and DNase-treated viruses.

The putative *Bacteroides* phage contig (D5PEG-A) represented only ~10% of reads in experimental D5 bioreactors before perturbation (Fig. 3A). Following perturbation with live D5 viruses, an immediate increase in the % reads mapped to the putative *Bacteroides* phage was observed (10.88% before perturbation to 56.49% within 1 h after perturbation). Reads aligning to this phage nearly saturated the D5-LV virome by day 15 (24 h after perturbation). Saturation also occurred in the D5-HTV ecosystem but transpired later on day 18 (4 days after perturbation). Assuming the heat-treated D5 bioreactor represents a replicable progression of an unperturbed D5 virome *in vitro*, this finding suggests Contig D5PEG-A is more representative of the late-stage D5 virome. While the same contig was briefly detectable in the D25A-LV virome immediately following its addition (0.11% to 25.27%), it could not be quantified in samples taken >24 h after perturbation and was never detected in the D25A heat-treated virome, suggesting the associated phage is not able to replicate in the D25A community.

Before perturbation, the putative *Clostridium* phage contigs (D5PEG-B and D5PEG-C) represented ~0.25% of viral reads present in the experimental D5 bioreactors (Fig. 3B). A small initial spike (0.28% before perturbation to 1.71% after perturbation) was detected in the D5-LV bioreactor. The same spike was absent in the heat-treated condition. Somewhat counterintuitively, whereas the signal remained <0.25% throughout the remainder of the experiment in the D5-LV condition, the signal rose to a peak of 20% on day 17 of the D5-HTV condition but vanished by the 20-day mark. This observation could be attributed to bacteriophages typically associated with a late-stage community, such as *Bacteroides*-associated contig D5PEG-A, saturating the community earlier in the D5-LV condition than is typical, preventing the emergence of more transient phage community members such as D5PEG-B/C.

In the D25A conditions before perturbation, alignments to the putative *Clostridium* phage contigs appeared to be relatively high compared to those in D5 (Fig. 3B, D25A live/heat), suggesting some phage(s) within D25A share gene content with contigs D5PEG-B and D5PEG-C. This shared gene content would confound the use of D5PEG-B and D5PEG-C as representatives of the D5 PEG-purified virome in D25A.

Across conditions, the D5 viruses introduced as perturbing agents were detectable against the native virome background via read mapping. Differences associated with heat treatment were more apparent in the short-term, up to 4 days after perturbation. Over longer timescales (4 to 14 days after perturbation), differences between communities perturbed with live and heat-treated phages were largely reduced. This suggests that the viromes were either resistant to the effects of perturbation, or that long-term virome composition was more driven by host population dynamics rather than extracellular virions alone.

To further analyze differences in viral communities between conditions, we generated a principal coordinate analysis (PCoA) plot, including all viral contigs >200 nucleotides in length for all experimental conditions. As expected, D5 viromes are separated from D25A viromes (Fig. 3D). The D5 vessel perturbed with live viruses appeared to follow a similar progression as the D5 vessel perturbed with heat-treated viruses, indicating that autochthonous perturbation did not have a substantial effect on the progression of viral D5 community composition. However, a substantial deviation was observed in the D25A bioreactor treated with live D5 viruses: a subset of D25A-LV virome samples are increasingly similar to D5 samples than D25A-HTV samples. This deviation appeared to be temporary because the D25A-LV samples later shifted and clustered with D25A-HTV samples. These results were consistent with the previous analysis of the percentage of reads mapped to the two most abundant perturbing phages (Fig. 3A to C), where a temporary spike in the percentage of reads mapped to D5 phages is observed in D25A-LV, but long-term relative abundances of viromes appear largely unchanged. Overall, this compilation of data suggested that intercommunity viral perturbation at least temporarily shaped virome contents, but that the observed differences were abrogated throughout the experiment.

### Alpha and beta diversity among bacterial communities.

**FIG 4:**
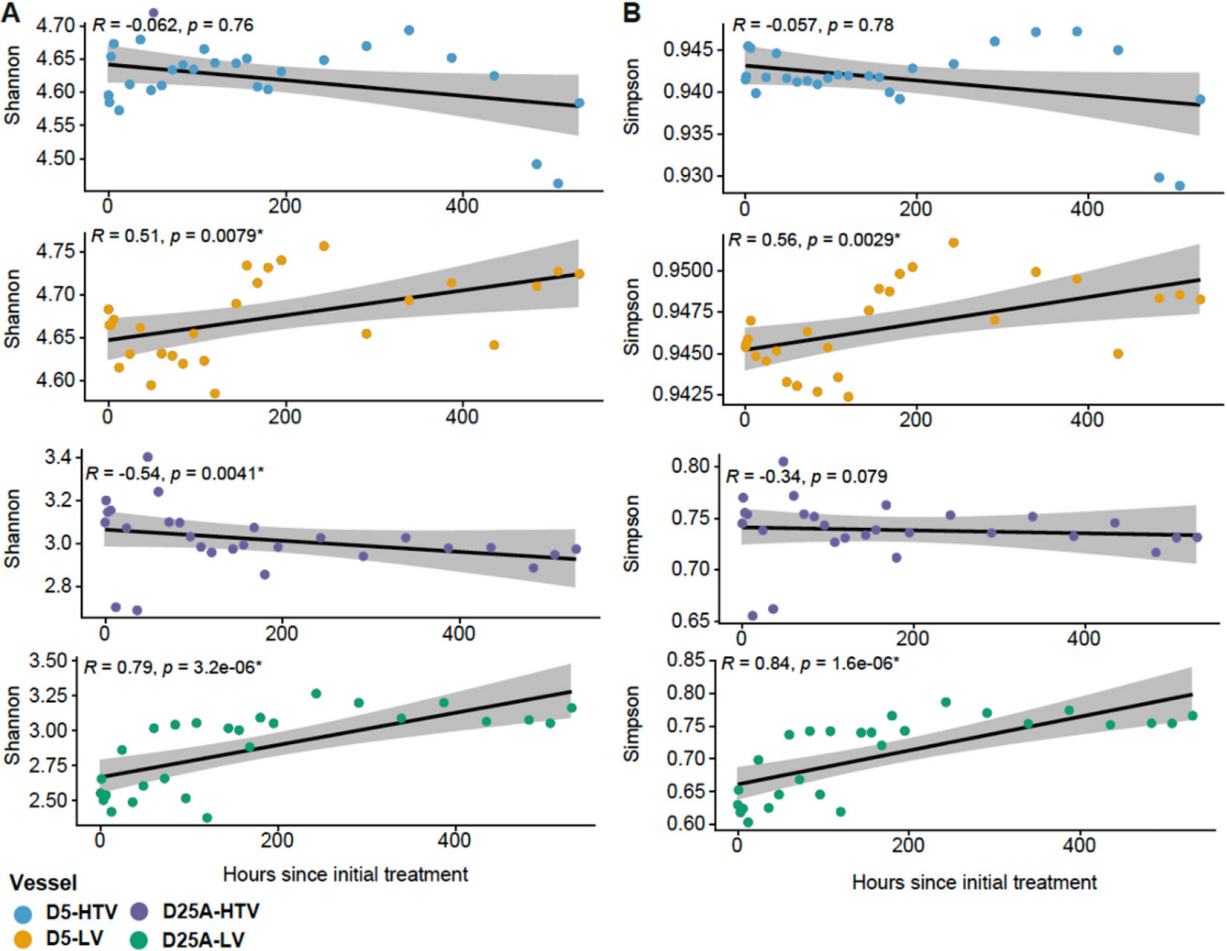
Scatterplot depicting the correlation of (A) Shannon and (B) Simpson with the number of hours after initial treatment with live or heat-treated viruses. The alpha diversity indices are shown on the *y* axis and the hours after initial treatment are depicted on the *x*-axis. The black line denotes the linear regression line with the gray shading indicating a 95% confidence interval. Spearman correlation indices and *P*-values are shown in the upper left corner of each panel. Points are colored by vessel type (D5-HTV, blue; D5-LV orange; D25A-HTV purple; D25A-LV green).

To determine whether treatment with live or heat-treated viruses was associated with changes in alpha diversity, we calculated two diversity metrics (Shannon and Simpson) as a function of time posttreatment. For both metrics, alpha diversity over time differed between live and heat-treated conditions (Fig. 4). We observed significant (Spearman; *P* < 0.05) positive correlations between diversity metrics and time posttreatment for all bioreactors treated with live viruses, whereas all bioreactors treated with heat-treated viruses displayed correlations in the negative direction. Of the negative correlations seen in bioreactors treated with heat-killed viruses, three were nonsignificant (D5A-HTV Shannon and Simpson, D25A-HTV Simpson) while D25A-HTV Shannon was significant (*R* = −0.54, *P* < 0.05).

**FIG 5:**
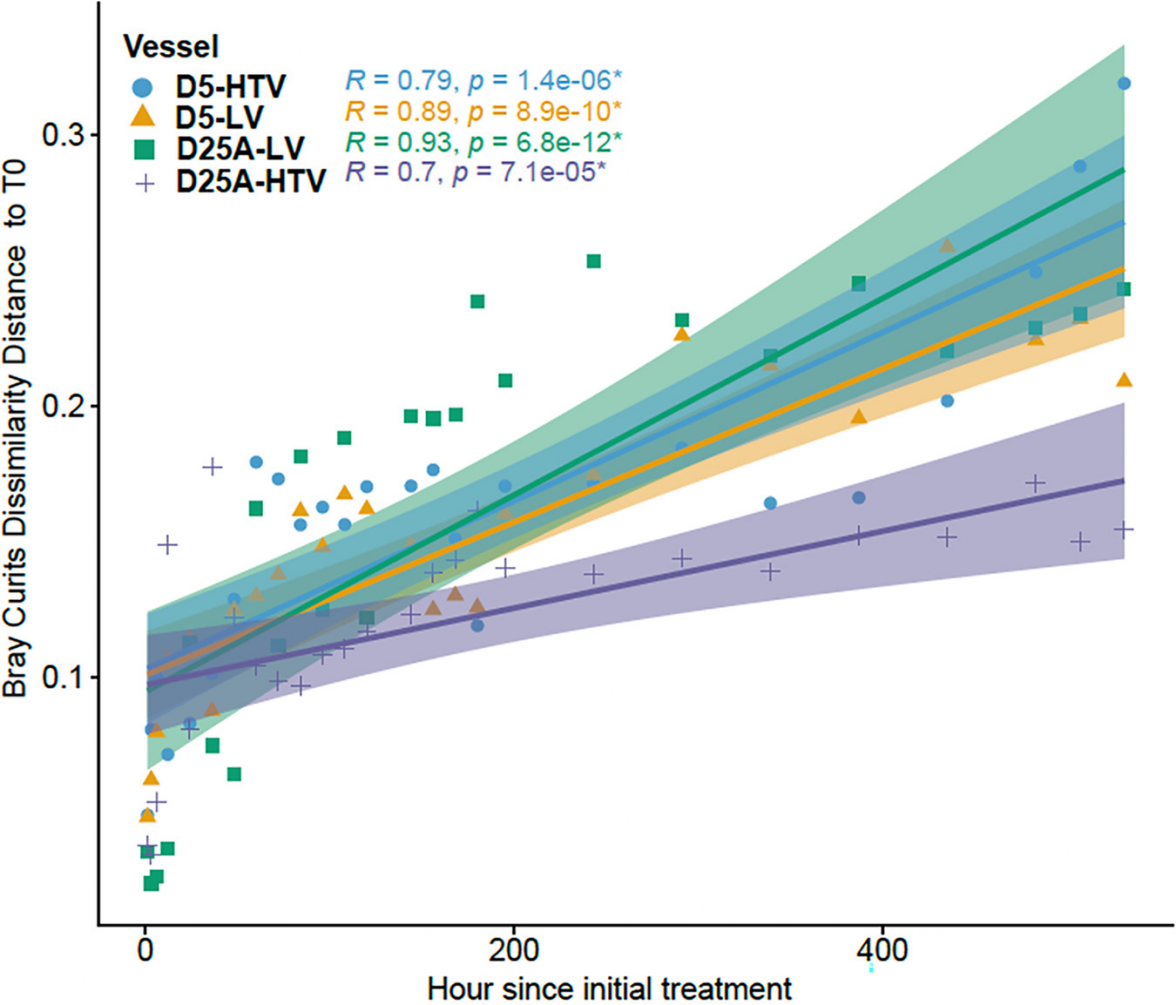
Scatterplot depicting the correlation between Bray-Curtis distance to time zero (T_0_) and the number of hours after initial treatment with live or heat-treated viruses. The Bray-Curtis distance to T_0_ is shown on the *y* axis and the hours after initial treatment are depicted on the *x*-axis. Color lines denote the linear regression lines for each vessel with the shading indicating a 95% confidence interval. Spearman correlation indices and *P* values are shown in the upper left corner. Points and lines are colored by vessel type (D5-HTV, blue; D5-LV orange; D25A-HTV purple; D25A-LV green).

As an alternative method to quantify shifts in community diversity following the addition of viruses, we calculated Bray-Curtis dissimilarity distances between the pretreatment sample (T0) and the samples taken posttreatment (Fig. 5). Bray-Curtis dissimilarity incorporates relative abundance data to estimate differences between community samples. We then calculated Spearman correlations between sample dissimilarity to T0 and each time point. We observed significant (Spearman, *P* < 0.05) positive correlations between Bray-Curtis dissimilarity and time for all bioreactor conditions (Fig. 5). However, for both D5 and D25A communities, we observed greater correlation coefficients between time and dissimilarity in bioreactors treated with live viruses compared to bioreactors treated with heat-treated viruses, although this distinction was not statistically significant (ANOVA, *P* > 0.05) (Fig. 5).

Additionally, we used Bray-Curtis dissimilarities as the input for principal coordinates analysis (PCoA) (Fig. S1). Here, we found clear, significant differentiation when comparing across communities (Fig. S1A; ANOSIM; *P* < 0.005). Sequences from each LV-treated bioreactor clustered significantly differently from those of the corresponding HTV-treated bioreactor of the same community (Fig. S1B and C; ANOSIM; *P* < 0.05).

### Temporal variations in bacterial taxonomic composition.

**FIG 6:**
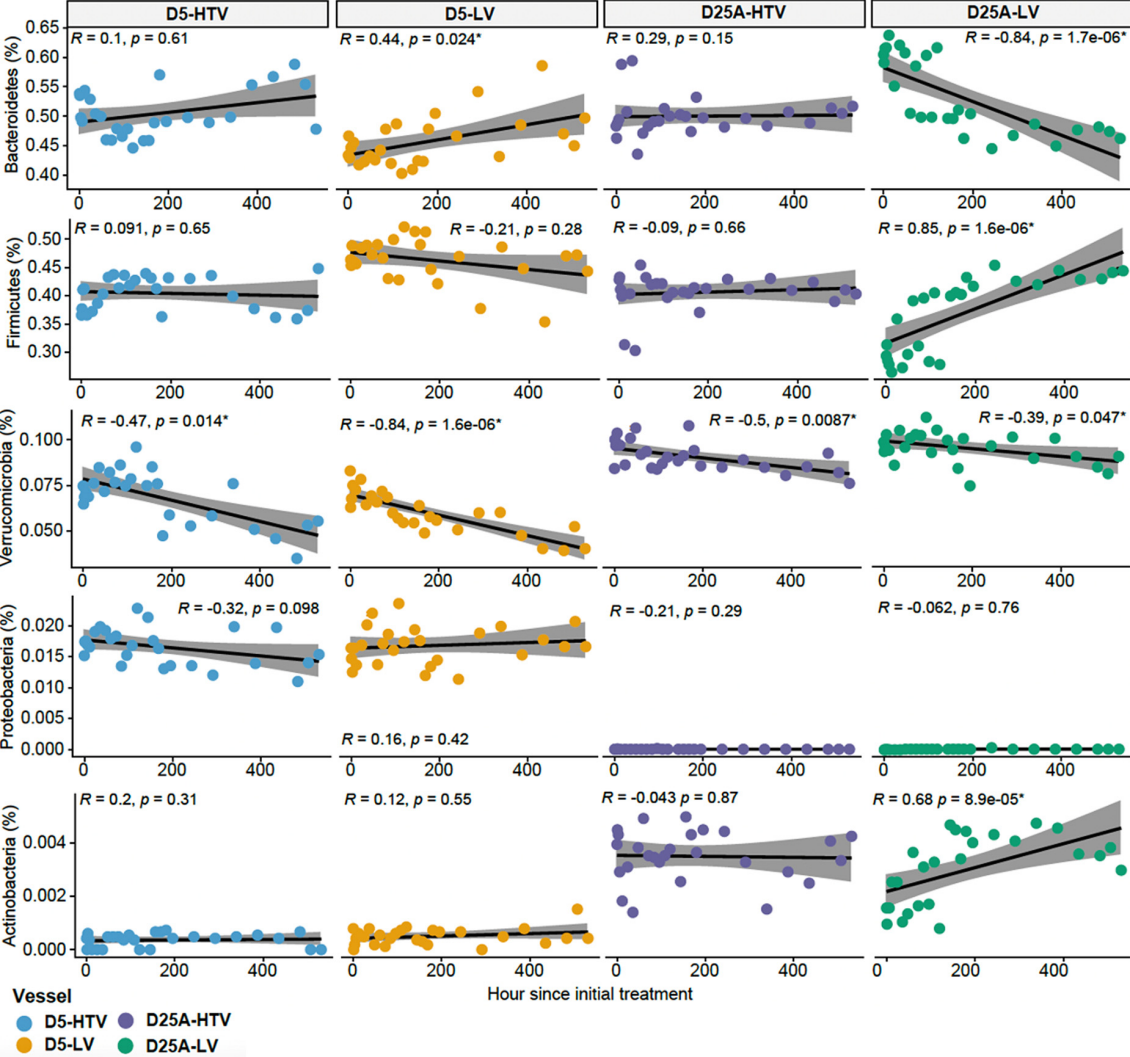
Scatterplot depicting the correlation between the relative abundance of the dominant bacterial phylum and the number of hours after initial treatment with live or heat-treated viruses. The relative abundance of phylum *Bacteroidetes*, *Firmicutes*, *Verrucomicrobia*, *Proteobacteria*, and *Actinobacteria* are shown on the *y*-axis, and the hours after initial treatment are depicted on the *x*-axis. The black line denotes the linear regression line with the gray shading indicating a 95% confidence interval. Spearman correlation indices and *P* values are shown in the corner of each panel. Points are colored by vessel type (D5-HTV, blue; D5-LV orange; D25A-HTV purple; D25A-LV green).

We next explored the relative abundances of bacterial genera present at ≥1% in a given sample for all bioreactors throughout the experiment (Fig. S2). In D5 bioreactors, *Bacteroides*, *Ruminococcus*, and *Akkermansia* were on average the three most abundant bacterial genera across live and heat-treated bioreactors, whereas *Alistipes*, *Akkermansia*, and *Eisenbergiella* were on average the three most abundant across D25A live and heat-treated bioreactors. Some trends were observed when examining relative abundances at the phylum level as a function of time posttreatment (Fig. 6). These trends were different depending on whether the bioreactors were perturbed with phage communities from the same subject (D5 bioreactor) or with phage communities from a different subject (D25 bioreactor). For example, intrasubject phage perturbations resulted in a significant increase in the phylum *Bacteroidetes* over time that was not observed after treatment with the heat-treated phages. However, there was a significant decrease in the representation of the phylum *Bacteroidetes* when intersubject perturbations were performed. Interestingly, the most abundant bacteriophage in the D5 PEG purified viromes used for perturbation is a putative *Bacteroides* phage (Fig. 3A), and we observe a stark decline in the abundance of *Bacteroides* in the D25A-LV but not D25A-HTV vessel following a perturbation (Fig. 6). However, causality cannot be stated without further investigation. A similar trend was also observed for the phyla *Firmicutes* and *Actinobacteria*, where intersubject perturbations were, but intrasubject perturbations were not, associated with significant changes in representation. Each of these trends was observed when the vessels were treated with live phages but was not observed in those vessels exposed to heat-treated phages.

**FIG 7:**
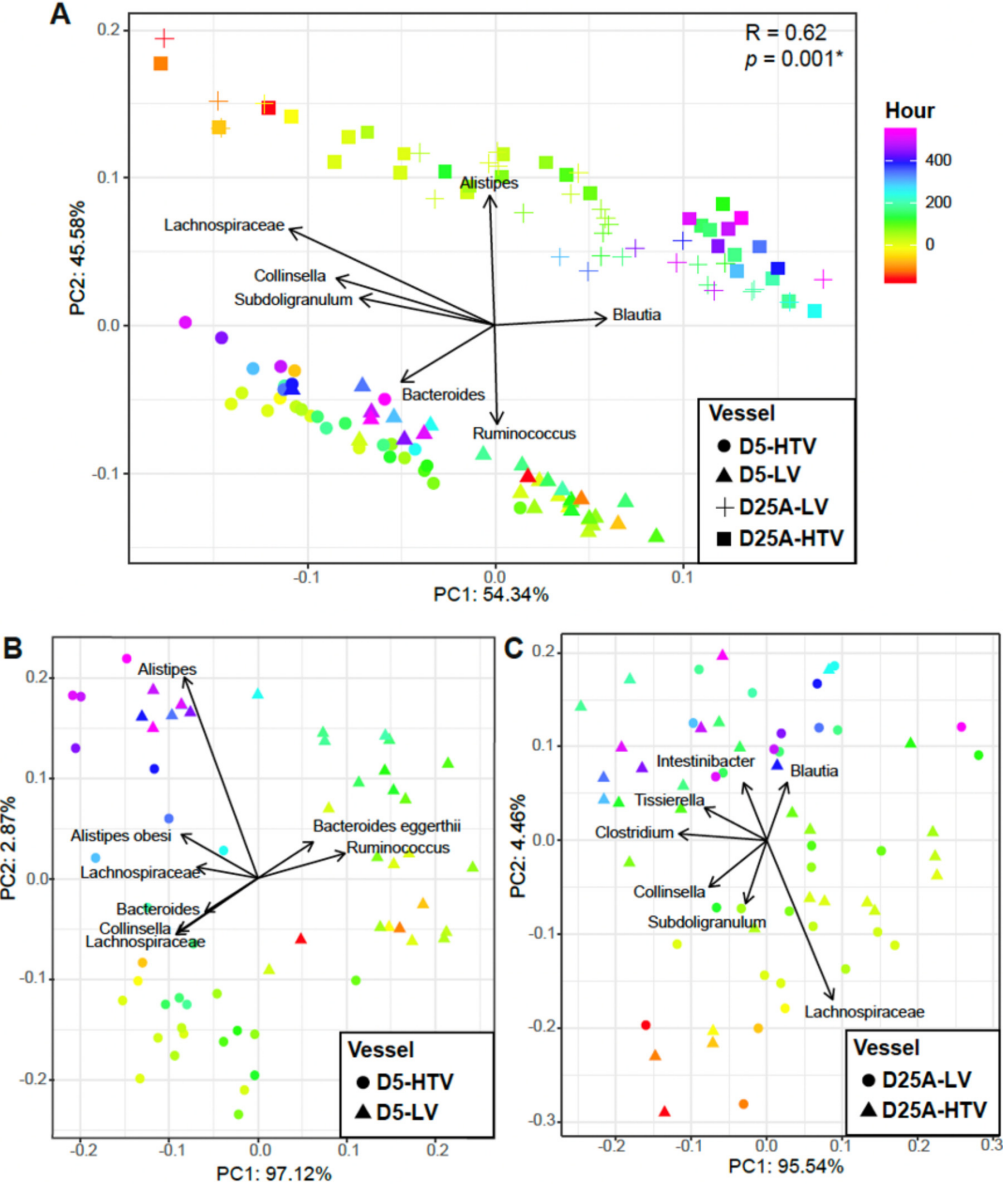
Aitchison compositional biplots for (A) all vessels and vessels broken up by (B) donor 5 and (C) donor 25A. Color denotes hour relative to the initial treatment with the live or heat-treated virus. Arrows denote important taxa with regard to sample clusters. Significance determined by ANOSIM with 999 permutations for vessel and denoted in the corner of each panel: *, *P* < 0.05.

To further assess which bacterial taxa were responsible for the observed beta diversity differences in the vessels in response to phage-mediated perturbations, we utilized Aitchison PCAs with overlaid biplots to identify the bacterial taxa that drive the sample clustering. As expected, we found that significant beta diversity differences were driving the separate clustering of samples from D5 and D25A bioreactors (Fig. 7A). The microbes responsible for driving these differences included *Alistipes* and *Blautia* for D25A samples, whereas *Ruminococcus* and *Bacteroides* were primarily responsible for distinguishing D5 samples (*R* = 0.62; *P* = 0.001 by ANOSIM). We further characterized differences that were observed in D5 (Fig. 7B) and D25A (Fig. 7C) bioreactors when each was perturbed with either live phages or heat-treated phages. For the D5 bioreactor that was perturbed with intrasubject phages, most of the differences were driven by microbes from the genera *Alistipes*, *Ruminococcus*, and Bacteroides eggerthii. For vessel D25A that was perturbed with intersubject phages, the differences observed were driven by several different microbes, including *Lachnospiraceae*, *Blautia*, *Tissierella*, *Intestinibacter*, *Clostridium*, *Collinsella*, and *Subdoligranulum*. The substantial differences in the changes observed between the two vessels treated with phage perturbations suggest that the live phage treatment was associated with distinct outcomes in microbial community composition, particularly when inter-subject perturbations were performed.

## DISCUSSION

In this study, we utilized chemostat bioreactors as *in vitro* models of the distal human colon and established a protocol for perturbing defined bacterial communities *in vitro* with bacteriophages purified from a separate defined microbial community. Our previous work has demonstrated that chemostat bioreactors support stable and reproducible microbial communities and succession (16, 17). Here, we demonstrated that foreign viruses were detectable against the autochthonous virome background and that alterations to bacterial community diversity and composition could be analyzed in relation to treatment condition and time posttreatment. We found that perturbation with intracommunity phages resulted in more rapid early saturation of a bacteriophage highly abundant in the late-stage community, but that virome compositions at later time points were largely similar between live and heat-treated conditions. This is consistent with a resilient succession of microbial strains and abundances toward a steady-state equilibrium, as observed in the maturation of other microbiomes (18). Perturbation with intercommunity phages was associated with short-term virome differences between live and heat-treated conditions. However, similar to the intracommunity perturbation, long-term outcomes appeared to be resilient to perturbation. That is, the virome of the D25A vessel perturbed with live viruses resembled that of the vessel perturbed with heat-treated viruses at later time points. This resilience to intercommunity phage perturbation could be attributed to limited host range, evolved host resistance, and/or noncompetitive efficiency of replication. Future studies involving the isolation of individual bacteriophages and bacterial community members, and subsequent exploration of host-range and replication efficiencies on intracommunity versus intercommunity hosts could help to clarify whether the differences between intracommunity and intercommunity phage perturbations seen in this study were driven by bacteriophage specificity or other population dynamics.

Prior studies probing phage-mediated perturbations of defined bacterial communities modeled intercommunity phage perturbations (1, 4). The current study is distinct in modeling both intercommunity and intracommunity phage perturbations. This distinction is especially relevant to understanding how native phage populations influence their associated gut bacterial communities over time, and how phages introduced from the local environment interact with this dynamic. In this study, we found greater impacts on bacterial community composition associated with intercommunity perturbation rather than intracommunity perturbation, though we would emphasize that this represents a single intercommunity pairing. We would also emphasize the observation of significant alterations to community composition in conditions treated with live phages, but not in conditions treated with heat-treated phages. As reported by Reyes et al. (1), we must infer that these outcomes were attributable to phage or phage-encoded factors, and cannot exclude that some nonphage, <0.45 μm, heat-mutable factor was causally responsible for the compositional changes observed. Nevertheless, the observed ‘live-phage’ outcomes are consistent with phage-host interactions, though the details of those interactions were not probed in this study.

One of the more interesting observations in this study is that disturbances to bacterial composition were observed regardless of whether intracommunity or intercommunity phage perturbations were performed. Intercommunity perturbations could be expected to trigger shifts in bacterial composition, especially if susceptible hosts have never previously encountered a particular phage and encode few mechanisms of defense. Understanding why intrasubject phage perturbations also altered bacterial composition is more difficult. One explanation involves the methodology for creating the D5 PEG-purified virus stocks used for intracommunity perturbation. These stocks were originally purified from a mixture of day 14 and day 21 bioreactor effluent. Because this multiple time point viral ‘cocktail’ was used to perturb experimental bioreactors on day 14, viral communities from later stages of community progression were introduced before they would naturally arise. Thus, the intracommunity perturbation performed could also be described as an intertemporal perturbation: an interaction between different periods of the same system. The specific mechanisms by which late-stage viruses shaped early-stage bacteria composition were not investigated in this study but could be probed in future work. For example, the complex communities used in the current study (68 and 77 strains for D5 and D25A, respectively) are stored as a library of isolated strains and could be reconstituted as isolates, subcommunities, or chimeric communities of D5 and D25A in future perturbation experiments. Isolation of gut phages for full experimental control presents a different challenge, although access to isolated bacterial strains presents a potential route for isolation of prophages, assuming a suitable prophage induction trigger could be verified. Alternatively, bacterial community members could be inoculated with purified viromes, and the phages subsequently plaque-purified and sequenced to identify phage-host linkages, in turn allowing analysis of community abundance relationships between the bacterial and viral metagenomes.

The major goal of this work was to establish a methodology for perturbing complex gut-associated bacterial communities with bacteriophages *in vitro*. Neither defined bacterial communities isolated from feces (19) nor the use of chemostat bioreactors to model gut microbiomes (20, 21) are novel approaches in microbiome experiments, but their use in tandem presents a new venue for gut phage-bacteria research. Chemostat bioreactors inoculated with feces have been known to support robust phage communities (17), however, the data presented here indicate that bioreactors inoculated with defined bacterial communities are also capable of supporting phage communities and that those communities are responsive to experimental manipulation.

The effects of phages on human microbial communities are relevant to human health and, thus far, have been understudied. While many have suggested that the presence of these diverse phage communities (3, 14, 22) may have significant impacts on resident microbial communities, there remains little in the literature thus far to support such a notion. Previous work has shown that phages can be readily shared in genetically related individuals (23), through breastfeeding (24), and through close contact, such as between roommates (25), yet we know very little about the impacts of such sharing events or the capacity of new phages to significantly shape human microbial communities. The model system presented here was developed to mimic the microbial community (including bacteria and phages) of the distal colon, and, as we demonstrated, can serve as a vessel for phage transfers. While our data have only characterized the effects of intersubject and intrasubject phage perturbations in the model systems of two human subjects, it has a substantial capacity to be adapted for multiple different human subjects to help define the parameters of phage-bacteria interaction in the gut. Given the significant results found in this study, where multiple microbial lineages were disturbed by live phages, we believe this model system affords the potential to define the role of individual phages as well as diverse phage communities in our microbiomes.

## MATERIALS AND METHODS

### Creation of defined bacterial communities.

Two defined bacterial communities were created to examine outcomes of phage-mediated perturbation on bacterial community dynamics *in vitro*. Both communities were constructed from a library of strains cultured from donor stool. The description of donor D5 and the original bacterial strain isolation procedure is described in Yen et al. (17), where D5 is referred to as ‘Donor A.’ Before use in the current study, 77 strains from Donor 5 were restreaked to purity from previously isolated frozen stocks, and their identity was verified by sequencing of the 16S rRNA V3 to V6 region. D25A strains were isolated from the stool of a healthy, 22-year-old female donor (26) D25A strains were isolated using methods described in (21). Briefly, donor stool was mixed in a saline buffer and a dilution range of 10^−4^ to 10^−8^ plated on various media types. Isolated colonies were picked and streaked to purity three times. Taxonomy was identified by Sanger sequencing of the 16S V3 to V6 region. Isolated strains were frozen down in skim milk freezing media (12% wt/vol skim milk powder, 1% vol/vol glycerol, 1% vol/vol dimethyl sulfoxide) as purified, single-strain stocks. Together, all single-strain stocks isolated from D25A feces comprised the D25A-defined community.

To prepare inoculum aliquots of each defined community, the purified bacterial isolates, originally isolated from donor feces, were individually cultured on Fastidious Anaerobe Agar (Lab M Ltd. Heywood, Lancashire, UK) supplemented with 5% defibrinated sheep’s blood (Hemostat Laboratories, Lancashire, UK) under anaerobic conditions. Isolates were restreaked to purity and strain identity reverified by Sanger sequencing of the 16S V3 to V6 region using BigDye reagents. Isolates specific to each respective community were then pooled in 5 mL prereduced sterile 0.9% saline. The saline suspension was homogenized by gently pipetting up and down, then transferred to 95 mL prereduced skim milk freezing media (12% wt/vol skim milk powder, 1% vol/vol glycerol). This was gently homogenized by bench-top swirling and inversion inside an anaerobic chamber. Five mL aliquots were then prepared in 15 mL centrifuge tubes. Each tube was sealed with Parafilm, removed from the anaerobic chamber, and flash-frozen in an ethanol-dry ice bath within 30 min. Aliquots were stored at −80°C until used as the inocula.

### Bioreactor operation.

Five hundred microliters Multifors bioreactor vessels (Infors AG, Bottmingen/Basel, Switzerland) were inoculated with aliquots from defined communities. Aliquots stored at −80°C were transferred to an anaerobic chamber and allowed to thaw at room temperature, after which they were gently inverted several times to mix. One aliquot was used to inoculate up to four bioreactor vessels with the same community. Bioreactor systems operations, including medium composition, have been previously described (16). Briefly, bioreactor parameters were set to mimic the physiology of the human colon: pH was held at 7.0, the temperature at 37°C, and anaerobic conditions were maintained through sparging with N_2_ gas. Feed medium was added to the vessel at a constant rate of 500 mL per vessel per day to achieve a 24 h retention period, and vessel contents were stirred to ensure homogenization. The experimental approach is depicted in Fig. 1.

### Isolation of virus-like particles (VLPs) from bioreactor effluent.

To produce stocks of D5 phages for use as perturbing agents, four bioreactor vessels were used to ‘farm’ D5 phages. Bioreactors were inoculated from a single D5 aliquot, allowed 14 days to equilibrate, and a 200 mL aliquot of vessel contents was harvested (40% culture volume). Vessels were then topped up with media, allowed a further 7 days to equilibrate, and another 200 mL harvested. Samples were then centrifuged at 5,000 × *g* for 30 min and the supernatant was passed through a 0.8 μm syringe filter followed by a 0.45 μm syringe filter. To ensure sterility, filtrates were filtered a second time through a 0.45 μm filter. Subsamples were cultured on chocolate agar plates and in thioglycolate broth for 28 days at 37°C to further ensure their sterility. No live bacteria could be recovered from these specimens. The presence of virus-like particles (VLPs) in D5 bioreactor effluent was verified using epifluorescence microscopy as in Patel et al. (27). To precipitate virions, 40 mL aliquots of filtrates were mixed with 10% wt/vol sterile polyethylene glycol (PEG-6000) and NaCl added to a final concentration of 0.5 M. Samples were incubated overnight while stirring at 4°C. The following day, samples were centrifuged at 5,000 × *g* for 20 min at 4°C. The pellet was resuspended in 2 mL SM buffer, vortexed, and mixed with 2 mL chloroform. Samples were then vortexed for 30 sec before being centrifuged at 2,500 × *g* for 5 min at room temperature. The aqueous top layer was collected and stored at 4°C.

The purified viruses were pooled, and 3 mL was removed from this pool for viral metagenomic sequencing. The remainder was then divided into four 25 mL aliquots. Two of the four aliquots were heat-treated by incubation at 95°C for 1 h, followed by autoclaving at 121°C for 20 min. Autoclaved samples were topped up to 25 mL with 0.02 μm-filtered water (Anotop 10 syringe filter, Whatman) to account for evaporation and were then treated with DNase for 1 h (1000 units of DNase I, Thermo Scientific, EN0521).

### Introduction of extracted bacteriophages into bioreactor vessels.

Fourteen days after inoculation with defined bacterial communities, bioreactors were perturbed with the live or heat-treated phage stocks derived from D5. To minimize oxygen introduced to bioreactors during perturbation, phage stocks were prereduced in an anaerobic chamber for 1 h on lab armor beads (Thermofisher) previously refrigerated at 4°C. Stocks were drawn into a 10 mL syringe, which was sealed and removed from the anaerobic chamber. Before introducing 25 mL phage stocks to each bioreactor vessel, 25 mL of the culture was first removed. Phage stocks were injected into respective vessels within 15 min of being removed from the anaerobic chamber (~10^10^ VLPs), either live or heat-treated, per vessel.

### Sample collection and processing.

A schematic of sampling time points is included in Fig. 1C. Each vessel was sampled daily with the following exceptions: on day 14, vessels were sampled 1h, 3h, 6h, and 12h after perturbation, after which vessels were sampled twice daily for 1 week, then daily for the last 2 weeks of the experiment. Samples were collected by aseptically removing 12 mL of culture directly from the bioreactor contents at approximately the 250 mL (half working volume) mark. To avoid overly altering the chemostatic conditions of the bioreactor, the daily total sampling volume did not exceed 10% of the total working volume in the vessel. For this reason, the sampling volume was reduced to 10 mL for each of the five sampling time points on day 16. Following collection, samples were immediately stored at −80°C and thawed at room temperature before use in subsequent analyses. A schematic of which time points were analyzed by 16S rRNA sequencing and virome metagenomic sequencing is included in Table S2.

### Transmission electron microscopy.

Virions were purified using a two-layer CsCl gradient, as in reference (28). A sample of phage concentrate from D5 bioreactor effluent (collected following PEG precipitation) was brought up to a final volume of 14 mL with SM buffer. Sample density was adjusted to 1.39 g/mL and loaded above 2 mL 1.7 g/mL CsCl. Samples were then ultracentrifuged at 150,000 × *g* for 4.5 h at 4°C. The density fractions between 1.41 and 1.50 g/mL were recovered and stored at 4°C. Before TEM imaging, 1 mL of the recovered phage suspension was centrifuged at 25,000 × *g* for 1 h at 4°C, after which 0.9 mL of supernatant was removed, and pelleted phages resuspended by gentle pipetting. A 5 μL droplet of the phage suspension was placed onto a glow-discharged copper grid with carbon-coated Formvar film and incubated for 30 s at room temperature. The excess solution was drained away on filter paper. Grids were incubated with 1% uranyl acetate for 10 s. Phage particles were viewed on an FEI Tecnai G2 F20 transmission electron microscope (FEI Company, Eindhoven, the Netherlands) and a Gatan Ultrascan 4k CCD camera (Gatan, Pleasanton, CA, USA).

### 16S rRNA gene sequencing.

Samples were thawed on ice (0.5 mL) and processed using the DNeasy PowerSoil kit (Qiagen) using the manufacturer’s instructions. The 16S rRNA V3-V4 gene region was amplified from microbial genomic DNA using forward primer 5′-TCGTCGGCAGCGTCAGATGTGTATAAGAGACAGCCTACGGGNGGCWGCAG-3′ and reverse primer 5′-GTCTCGTGGGCTCGGAGATGTGTATAAGAGACAGGACTACHVGGGTATCTAATC-3′. DNA was amplified with KAPA HiFi HotStart ReadyMix (Roche), followed by a cleanup step using AMPure XP beads (Beckman). Illumina Nextera XT adapter sequences (Illumina) were added using KAPA HiFi HotStart ReadyMix (Roche) and cleaned by using AMPure XP beads (Beckman). DNA concentration was determined using a Qubit (ThermoFisher Scientific) and the size was analyzed using a Bioanalyzer (Agilent). Samples were diluted to 4 nM using 10 nMol Tris, pH 8.5, and sequenced on the Illumina MiSeq platform.

### Analysis of 16S rRNA gene sequences.

16S rRNA sequence reads were quality filtered and dereplicated using the DADA2 plugin in Quantitative Insights into Microbial Ecology 2 (\2; version 2019.7) (28, 29). Alpha (observed operational taxonomic units [OTUs], Shannon index, and Simpson) and Beta (Bray-Curtis dissimilarity, Jaccard, weighted Unifrac) diversity metrics were produced by QIIME2 core-metrics-phylogenetic pipeline (sampling-depth parameter 16,500). Robust Aitchison PCA ordination was calculated with the DEICODE plugin with taxonomic biplot overlays (30). Taxonomic classifications were generated using the QIIME feature-classifier classify-sklearn feature, with a naïve Bayes classifier trained on the SILVA database and visualized at the phylum and genus levels (31). Data were visualized using the qiime2R, ggplot2, and pheatmap packages in R-Studio (version 1.0.153) (32–34).

Beta-diversity significance was determined using ANOSIM tests with 999 permutations. Spearman correlation coefficients were calculated to identify associations between the hours after initial treatment and the alpha diversity metrics (observed OTUs, Shannon index, and Simpson), Bray-Curtis dissimilarity distance to T0, and the relative abundance of the dominant bacteria phyla. Analysis of covariance (ANCOVA) with Bonferroni correction was also used to compare the regression lines calculated between the hours after initial treatment and Bray-Curtis distance to time zero (T_0_). Alpha diversity comparisons between bioreactors were assessed by ANOVA, and *post hoc* Tukey’s honest significant difference (HSD) tests were conducted to correct for multiple comparisons. All statistical analyses were conducted in R Studio (version 1.0.153).

### Viral DNA shotgun sequencing.

Viral DNA extraction method was adapted from Conceição-Neto, et al. (35). One milliliter bioreactor effluent samples were thawed on ice and placed on the Minilys homogenizer for 1 min at 2000 rpm. Samples were centrifuged at 17,000 × *g* for 3 min, and 300 μL supernatant was then filtered through a 0.8 μm PES filter at 17000 × *g* for 1 min. Samples were then treated with nucleases by adding 4 μL benzoase, 2 μL micrococcal nuclease, and 14 μL 20× buffer (1 M Tris, 100 mM CaCl_2_, and 30 mM MgCl_2_, pH 8.0). After mixing by inversion, samples were incubated at 37°C for 2 h. The reaction was stopped by adding 14 μL 10 nM EDTA. Samples were added to an Amicon Ultra-4 (Millipore) centrifugal filter with a cutoff of 10,000 MWCO and centrifuged at 4,000 × *g* for 10 min using a swinging bucket rotor. The retentate was collected and nucleic acid extraction was performed using the QIAamp Viral RNA Minikit (Qiagen).

Amplification was performed by modifying the Complete Whole Transcriptome Amplification Kit Protocol (WTA2) (Sigma-Aldrich). To capture low abundance viral sequences, the WTA2 kit was modified by increasing the number of amplification cycles to 22. Following this protocol, the nucleic acid yield was measured by using the Qubit dsDNA HS assay kit (ThermoFisher Scientific). Library preparation was performed using the Nextera XT kit (Illumina), with 1.2 ng/μL as the input material. The Nextera XT protocol was modified by decreasing the tagmentation time from 5 to 4 min to favor larger DNA fragments, and the PCR extension time was increased to 45 s. Samples were purified following PCR using AMPure beads and the library size was checked with a Bioanalyzer (Agilent). Libraries were pooled into a 2 nM solution, denatured, and sequenced on an Illumina MiSeq platform (Illumina) using the MiSeq reagent kit v3 (Illumina).

### Analysis of viromes.

Virome reads were analyzed according to a modified version of the protocol described in Santiago-Rodriguez, et al. (17). Reads were trimmed using CLC Genomics Workbench (CLC Bio USA, Cambridge, MA), removed low complexity reads with ≥8 consecutive homopolymers and removed reads with substantial length variation (<100 nucleotides or >200 nucleotides). Reads were then mapped to common bacterial and human contaminants using BLASTN (E value <10^−5^) against the Ribosomal Database Project 16S rRNA genes database (36) and the human reference database available at https://www.ncbi.nlm.nih.gov/genome/guide/human/. Reads with significant similarities to human or common bacterial contaminant sequences (74,011 of 26,721,717 [0.28%] reads) were removed before assembly (Table S3). Reads were then assembled using the Metagenomics *de novo* assembly module in CLC Genomics Workbench with 98% identity and a minimum 50% read overlap. Consensus sequences were constructed according to the majority rule, and any contigs with ambiguous characters or lengths <200 nucleotides were removed before further analysis. Virome contigs were annotated using BLASTx against the NCBI nr database with an E value cutoff of 0.001. Results were parsed using the Ion Assist program for viral genes with an E value cutoff of <10^−5^.

### Data availability.

All sequences included in this study have been deposited in the NCBI Sequence Read Archive. 16s rRNA sequencing is available under BioProject accession PRJNA779730, and virome sequencing is available under BioProject accession PRJNA779729.
