# Supplementary figures and images for: Bacteriophage-Mediated Perturbation of Defined Bacterial Communities in an In Vitro Model of the Human Gut
Source: Microbiol Spectr. 2022 May 31;10(3):e01135-22. doi: 10.1128/spectrum.01135-22 (PMC9241613; doi:10.1128/spectrum.01135-22)

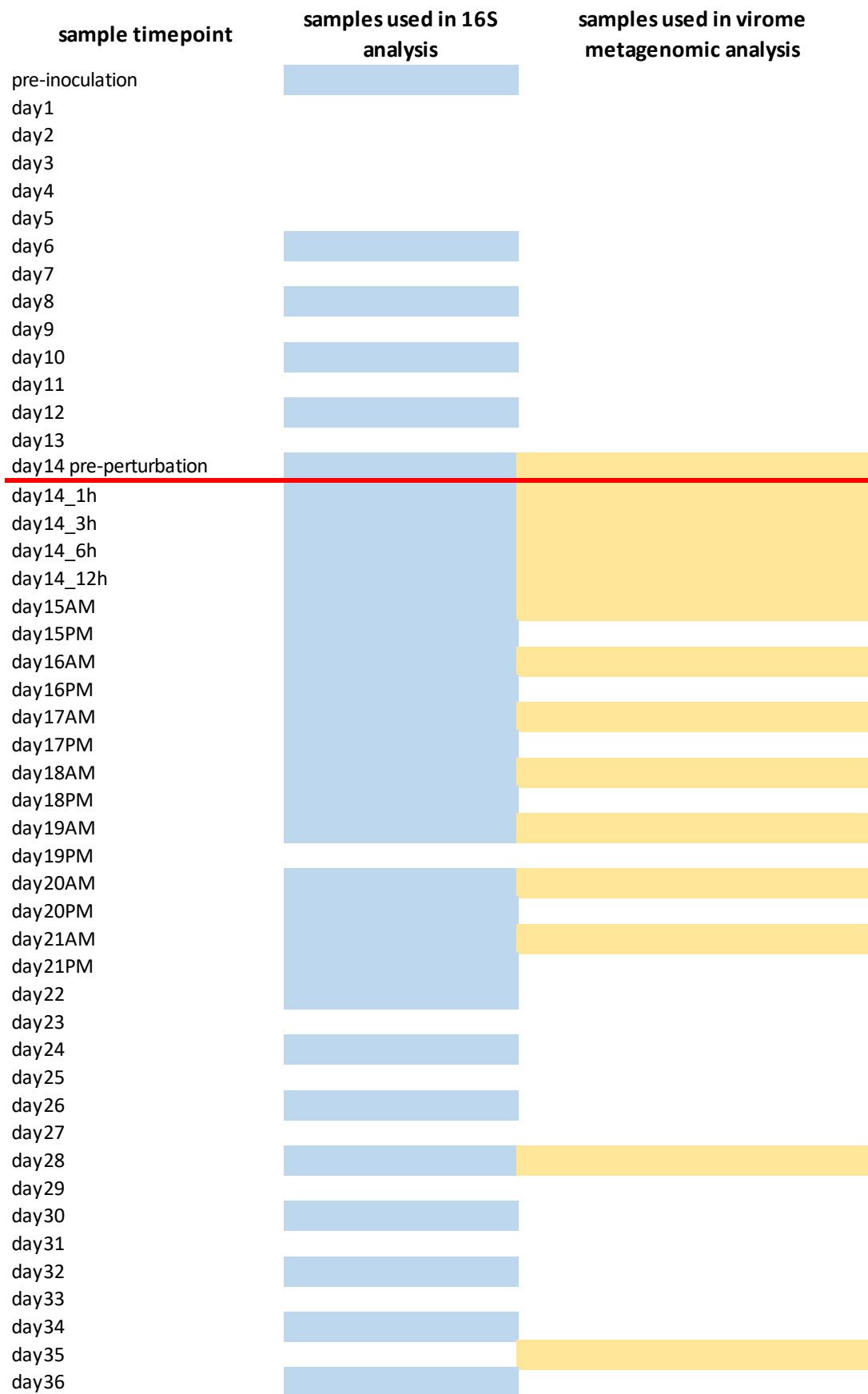

Perturbation with live or heat-treated viruses represented as a red line.

Supplement: Supplemental file 3 — Supplemental material. Download spectrum.01135-22-s0003.pdf, PDF file, 0.04 MB [file spectrum.01135-22-s0003.pdf]
